# Thermal-fluid-structure coupling simulation of filling process of storage tanks with ultra-low temperature and high-flow-rate

**DOI:** 10.1038/s41598-026-49937-7

**Published:** 2026-04-20

**Authors:** Zhao Zhang, Tianyu Chen, Jingyuan Chen

**Affiliations:** Department of Engineering Mechanics, Dalian University of Technology, Dalian, 116024 China

**Keywords:** Thermal-fluid-structure coupling, Simulation, Storage tank, Ultra-low temperature, High-flow-rate, Energy science and technology, Engineering, Materials science

## Abstract

To investigate the tank deformation behavior under ultra-low temperature and high-flow-rate filling conditions, a thermal-fluid-structure coupling numerical model for liquid hydrogen (LH_2_) filling into the storage tank was established. The temperature variation and corresponding structural deformation of the tank during the ultra-low temperature high-flow-rate LH_2_ filling process were systematically analyzed. The research findings reveal that at a filling flow rate of 8 m^3^/min and a pressure difference of 0 MPa, 84.1% of the tank volume was filled with fuel within 960 s, resulting in a deformation of 30.418 mm. When the outlet pressure difference increased from 0 to 0.1 MPa, the fuel filling ratio reached 78.5% in 500 s, with a corresponding deformation of 28.907 mm. As the outlet pressure difference further increased from 0.1 to 0.2 MPa, the filling ratio decreased to 45% with a filling duration of 576 s, and the deformation was reduced to 24.527 mm. When the filling flow rate was increased to 15 m^3^/min, 44% of the tank volume was filled in 250 s, with a deformation of 27.043 mm. Comparative analysis demonstrates that high-flow-rate LH_2_ filling achieves significantly higher efficiency than low-flow-rate filling, while the structural deformation induced by high-flow-rate filling is larger than that by low-flow-rate filling. When pre-cooling measures were adopted, the tank deformation after switching to high-flow-rate filling was notably smaller than that without pre-cooling. It is therefore concluded that pre-cooling measures are essential for ultra-low temperature high-flow-rate LH_2_ filling, as they can significantly improve the LH₂ filling efficiency while effectively reducing the thermal deformation of the tank structure.

## Introduction

Cryogenic fuel tank filling is a crucial link in space launch missions and an important prerequisite for ensuring the stable, efficient, and safe operation of the entire space launch mission^1–3^. Cryogenic propellants represented by liquid hydrogen (LH₂) and liquid oxygen (LO₂) are widely used in space missions due to their high specific impulse characteristics^4^. As the filling temperature decreases, the density of liquid fuel increases accordingly. Taking liquid hydrogen as an example, data show that the density of saturated liquid hydrogen at 22 K is 68.745 kg/m³ (0.1635 MPa), and when the temperature drops to 14 K, the density of saturated liquid hydrogen increases to 76.821 kg/m³ (0.007884 MPa). Moreover, with the increase of pressure, the density of liquid hydrogen will further increase, so that the tank with the same volume can store a larger mass of fuel, ensuring the smooth completion of space missions. With the development of space missions, the filling temperature of the tank is moving towards deep cryogenic temperature and the filling flow rate towards large flow, which makes the tank structure exhibit characteristics significantly different from conventional filling during the liquid fuel filling process. This new characteristic determines the necessity of carrying out thermal-fluid-solid coupling numerical simulation of liquid hydrogen filling into the tank under ultra-low temperature and large flow conditions.

From the perspective of filling environment, fuel filling is mainly divided into two categories: gravity-assisted filling and gravity-free filling, corresponding to different working conditions of ground filling and on-orbit filling. From the perspective of exhaust characteristics, fuel filling is mainly divided into vented filling and ventless filling. Among them, ventless top filling is mainly divided into three stages: initial rapid pressure rise, stable filling, and final rapid pressure rise^5-6^. Its filling process is affected by the thermodynamic characteristics of fuel and gravity. It is believed that compared with the gravity-assisted condition, ventless filling is expected to perform better under microgravity by the studies of the influence laws of filling port structure, initial wall temperature, filling temperature, filling flow rate and other conditions on the filling process through CFD (Computational Fluid Dynamics) technology simulation. By the studies of the influence of inlet flow rate, temperature, initial pressure and other factors on the thermodynamic behavior of liquid hydrogen filling based on the CFD-based multiphase flow model^7^, it is found that the increase of inlet temperature can lead to the increase of absolute pressure, enhance the trend of thermal evaporation, and reduce the efficiency of fuel filling. It is proposed^8,9^ that subcooled filling can be adopted to increase the density of liquid propellants and reduce the saturation pressure, thereby improving the thermodynamic performance of propellants. The temperature drop characteristics of liquid oxygen during the cycle cooling process was studied by means of simulation. The results showed that the temperature drop uniformity of the tank is good^10^.

It can be found from the above studies that the fuel filling process and technology can be simulated by methods such as CFD^11–15^, which have achieved good calculation accuracy and effect compared with experimental results^16–20^. However, the ultra-low temperature and large flow liquid hydrogen filling of the tank is a complex thermal coupling process^21^, which has a significant impact on the tank structure^22–26^. At present, there are relatively few studies on this problem. Therefore, this paper adopts the methods of thermal coupling and fluid-solid coupling to study the temperature change, gas-liquid phase change during the tank cooling process under the conditions of ultra-low temperature (14.6 K) and large flow (≥ 8 m³/min) liquid hydrogen filling, as well as the changes of mechanical behaviors such as tank structural deformation and stress caused thereby. The change mechanisms of thermal coupling and fluid-solid coupling during ultra-low temperature and large flow liquid hydrogen filling of the tank are summarized, which provides a theoretical reference for ultra-low temperature and large flow fuel filling of the tank.

## Models and methods

**Fig. 1:**
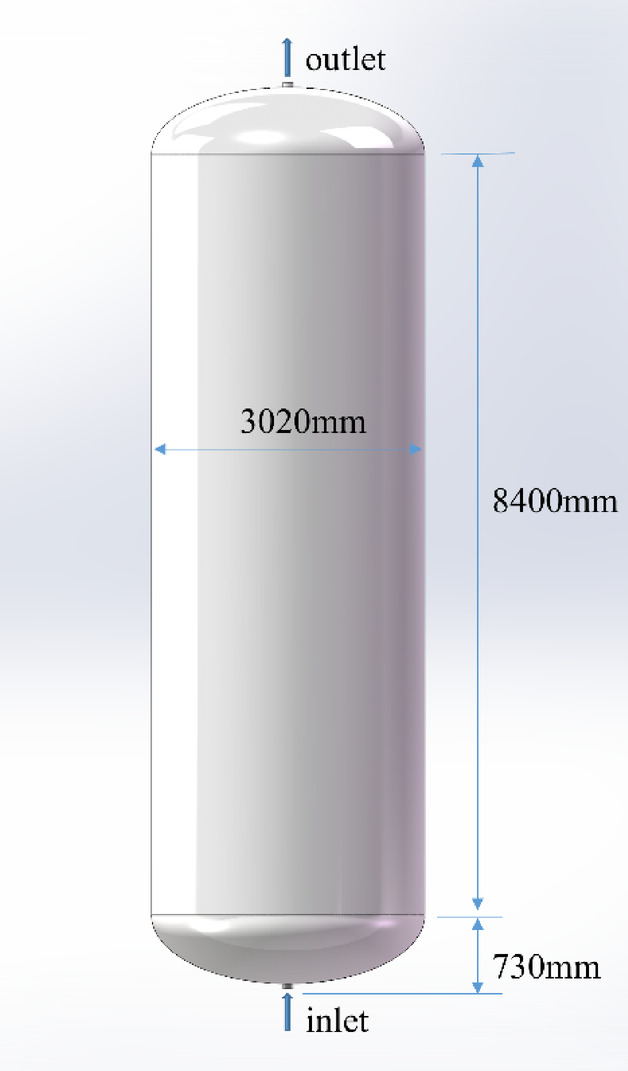
Geometrical model of tank.

**Fig. 2:**
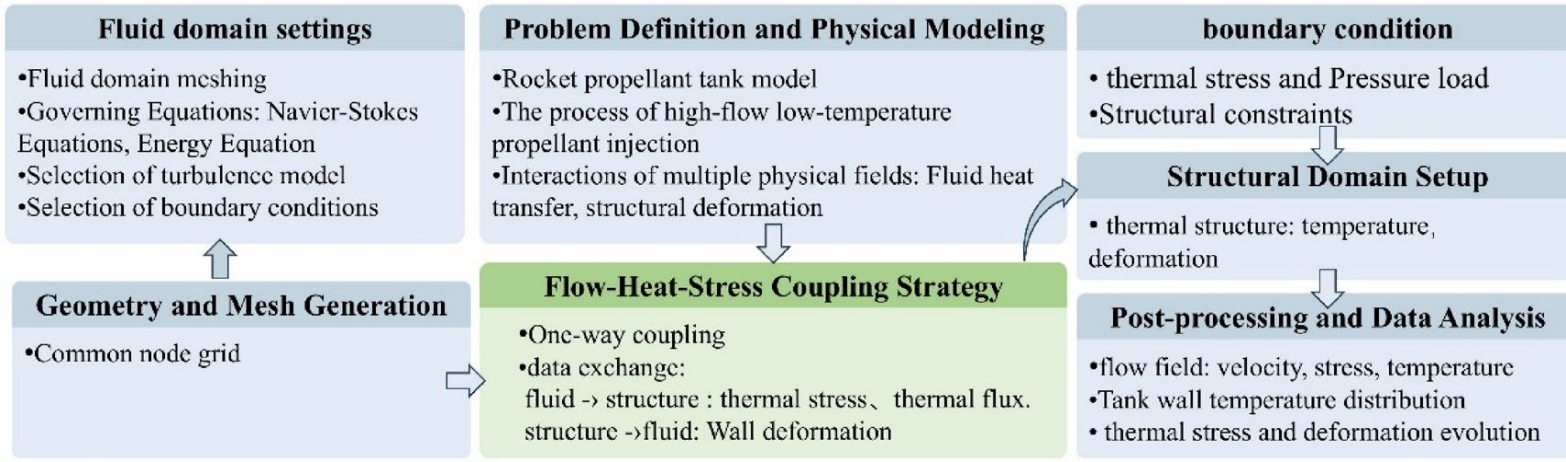
Fluid-solid thermal coupling flow chart.

As shown in Fig. 1, the established model consists of a cylindrical section and upper and lower ellipsoidal heads. The inlet and outlet ports each have an inner radius of 50 mm and a height of 60 mm. The cylindrical section has an inner radius of 1506 mm and a height of 8400 mm, with a head height of 730 mm and a wall thickness of 4 mm. The tank is filled with liquid hydrogen. In the first stage, air at 300 K is steadily injected into the tank to ensure that both the ambient temperature and the initial temperature are maintained at 300 K. In the second stage, the flow velocity is determined for different flow rates, and corresponding filling conditions are specified accordingly. The double-precision solver in Fluent was employed. The Volume of Fluid(VOF) multiphase flow model was selected to capture the gas-liquid interface distribution during the filling process. The k-epsilon model combined with enhanced wall treatment was used to analyze the turbulent effects of the fluid inside the tank. For the solution method, the Coupled algorithm was chosen. In terms of spatial discretization, the Gradient was set to Least Squares Cell Based, the Pressure term used the PRESTO! scheme, the Momentum equation adopted the Second Order Upwind scheme, the Volume Fraction term selected the Compressive scheme, and both the Turbulent Kinetic Energy and Turbulent Dissipation Rate terms used the First Order Upwind scheme. The flowchart is shown in Fig. 2.

For the fluid mechanics part of the filling process, computational fluid dynamics (CFD) is mainly used for calculation. The Navier-Stokes equations are adopted to describe the motion law of viscous fluids.

1 $$\:\rho\:\left(\frac{\partial\:u}{\partial\:t}+u\cdot\:\nabla\:u\right)=-\nabla\:p+\mu\:{\nabla\:}^{2}u+\rho\:g+{F}_{\mathrm{o}\mathrm{t}\mathrm{h}\mathrm{e}\mathrm{r}}$$

where ρ is the fluid density, u is the fluid velocity, p is the pressure, µ is the dynamic viscosity, and f is the body force. When the viscosity is small, the equations can be reduced to the Euler equations for ideal fluids.

The continuity equation describes the conservation of mass of the fluid. For incompressible fluids,

2 $$\begin{array}{*{20}c} {\nabla \cdot u = 0} \\ \end{array}$$

By performing the Reynolds decomposition on the instantaneous Navier-Stokes equations, and introducing the turbulent eddy viscosity, the Reynolds stresses are related to the gradient of the mean velocity via the Boussinesq hypothesis. This yields the classic k-ε model for closing the Reynolds stress terms, enabling the solution of the Reynolds-averaged Navier-Stokes (RANS) equations.

3 $$\begin{array}{*{20}c} {\frac{{\partial \left( {\rho k} \right)}}{{\partial t}} + \nabla \cdot \left( {\rho ku} \right) = \nabla \cdot \left[ {\left( {\mu _{t} + \frac{\mu }{{\sigma _{k} }}} \right)\nabla k} \right] + \mu _{t} \left( {\nabla u:\nabla u^{T} } \right) - \rho \varepsilon } \\ \end{array}$$

4 $$\begin{array}{*{20}c} {\frac{{\partial \left( {\rho \varepsilon } \right)}}{{\partial t}} + \nabla \cdot \left( {\rho \varepsilon u} \right) = \nabla \cdot \left[ {\left( {\mu _{t} + \frac{\mu }{{\sigma _{\varepsilon } }}} \right)} \right] + C_{{1\varepsilon }} \frac{\varepsilon }{k}\mu _{t} \left( {\nabla u:\nabla u^{T} } \right) - C_{{2\varepsilon \rho }} \frac{{\varepsilon ^{2} }}{k}} \\ \end{array}$$

where$\:\nabla\:\bullet\:\left(\rho\:ku\right)$ is the convection term of $\:\epsilon\:$, and $\:\nabla\:\bullet\:\left[\left({\mu\:}_{t}+\frac{\mu\:}{{\sigma\:}_{k}}\right)\nabla\:k\right]$ is the diffusion term of $\:\epsilon\:$.

The VOF method is adopted to simulate the gas-liquid phase change in the tank and track the multiphase flow interface between gas and liquid. The phase volume fraction transport equation is solved to accurately capture the dynamic position change of the gas-liquid interface. The phase volume fraction α is introduced.

5 $$\:\sum\:{\alpha\:}_{i}=1$$

where *i* represents different phase.

Solving the transport equation,

6 $$\:\frac{\partial\:\alpha\:}{\partial\:t}+\nabla\:\cdot\:\left(\alpha\:\mathbf{u}\right)=0$$

where **u** is velocity vector.

The fluid boundary acts as an applied load, which, together with the thermal load, constitutes the external load for solving the solid deformation. The heat transfer equation is as follows,

7 $$\begin{array}{*{20}c} {\rho c_{p} \left( {\frac{{\partial T}}{{\partial t}} + u \cdot \nabla T} \right) = k\nabla ^{2} T + \Phi } \\ \end{array}$$

where *T* is the temperature, *c*_p_ is the specific heat capacity, *k* is the thermal conductivity, *Φ* is the viscous dissipation term, and *u* is the fluid velocity. The solved temperature is applied as a boundary condition to the solid boundary for the thermo-mechanical coupling solution.

The heat conduction equation in the solid domain is,

8 $$\begin{array}{*{20}c} {\rho _{s} c_{s} \frac{{\partial T}}{{\partial t}} = \nabla \cdot \left( {k_{s} \nabla T} \right) + S_{s} } \\ \end{array}$$

Temperature is introduced as a boundary condition into the thermo-mechanical coupling equations for solution,

9 $$\begin{array}{*{20}c} {\nabla \cdot \sigma + f_{b} = 0} \\ \end{array}$$

10 $$\begin{array}{*{20}c} {\sigma = c:\left( {\varepsilon - \alpha _{s} \left( {T - T_{0} } \right)I} \right)} \\ \end{array}$$

The above models are established and solved using ANSYS.

## Mesh sensitivity

**Fig. 3:**
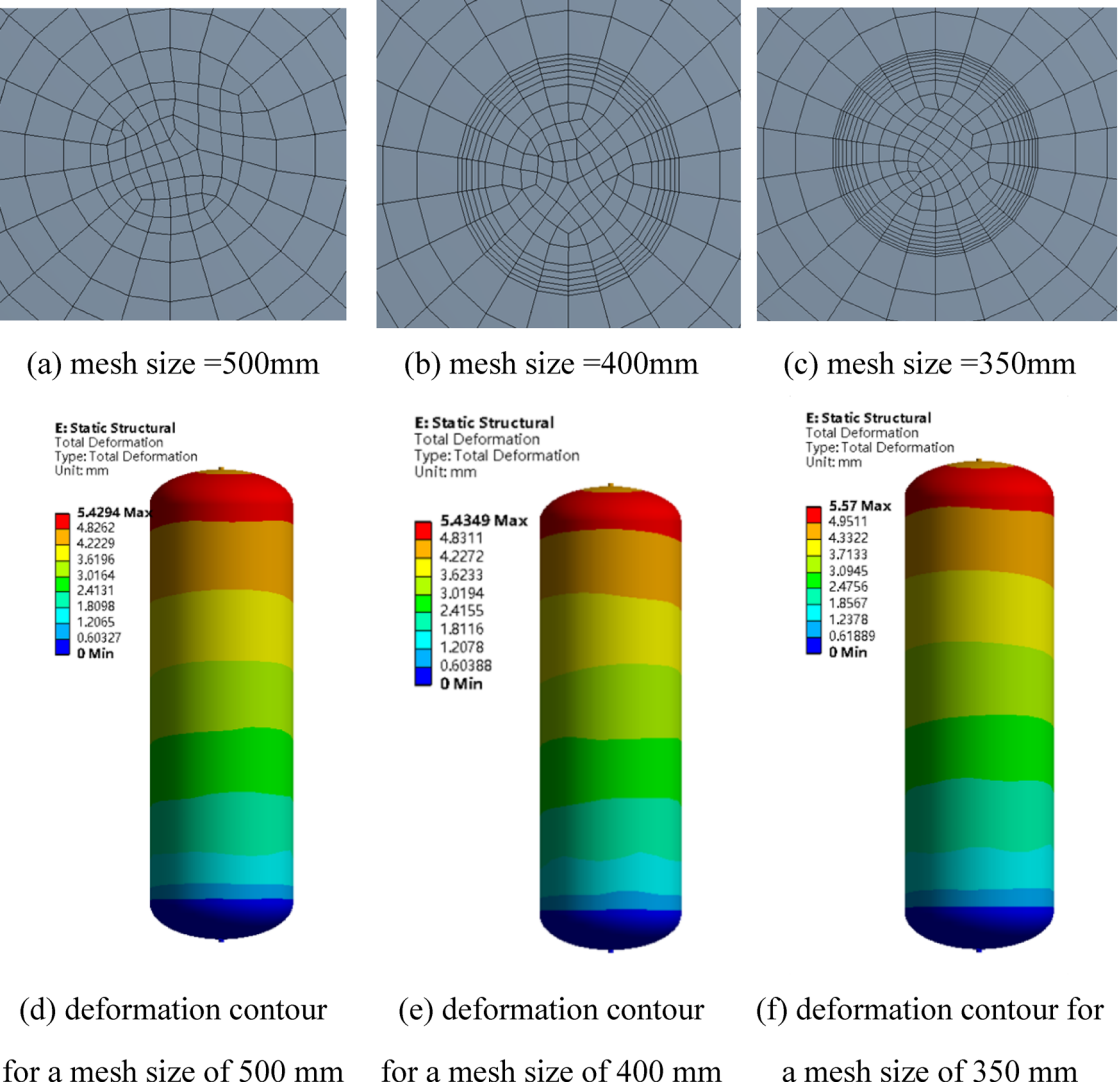
Mesh sensitivity analysis.

To demonstrate the effect of the mesh on the calculation results, a mesh dependency study was performed on the model shown in Fig. 1. The mesh sizes and corresponding deformation contours are shown in Fig. 3.Specifically, Fig. 3a has a mesh size of 500 mm, Fig. 3b has a mesh size of 400 mm, and Fig. 3c has a mesh size of 350 mm, with the locally refined mesh shown in the figures. A mesh independence calculation was conducted at a flow rate of 8 m³/min and an external pressure of 0.1 MPa. The deformation results at time t = 50 s were selected for comparison. From the comparison in Fig. 3, it was found that when the mesh size was 500 mm, its effect on the calculation results was limited. When the mesh size is refined from 400 mm to 350 mm, the maximum deformation increases by 2.5% ( less than 3% ), and the results have no obvious fluctuation, indicating that the numerical value is reliable. Considering the simulation computation time, a mesh size of 400 mm was chosen for the current model.

## Results and discussions

**Fig. 4:**
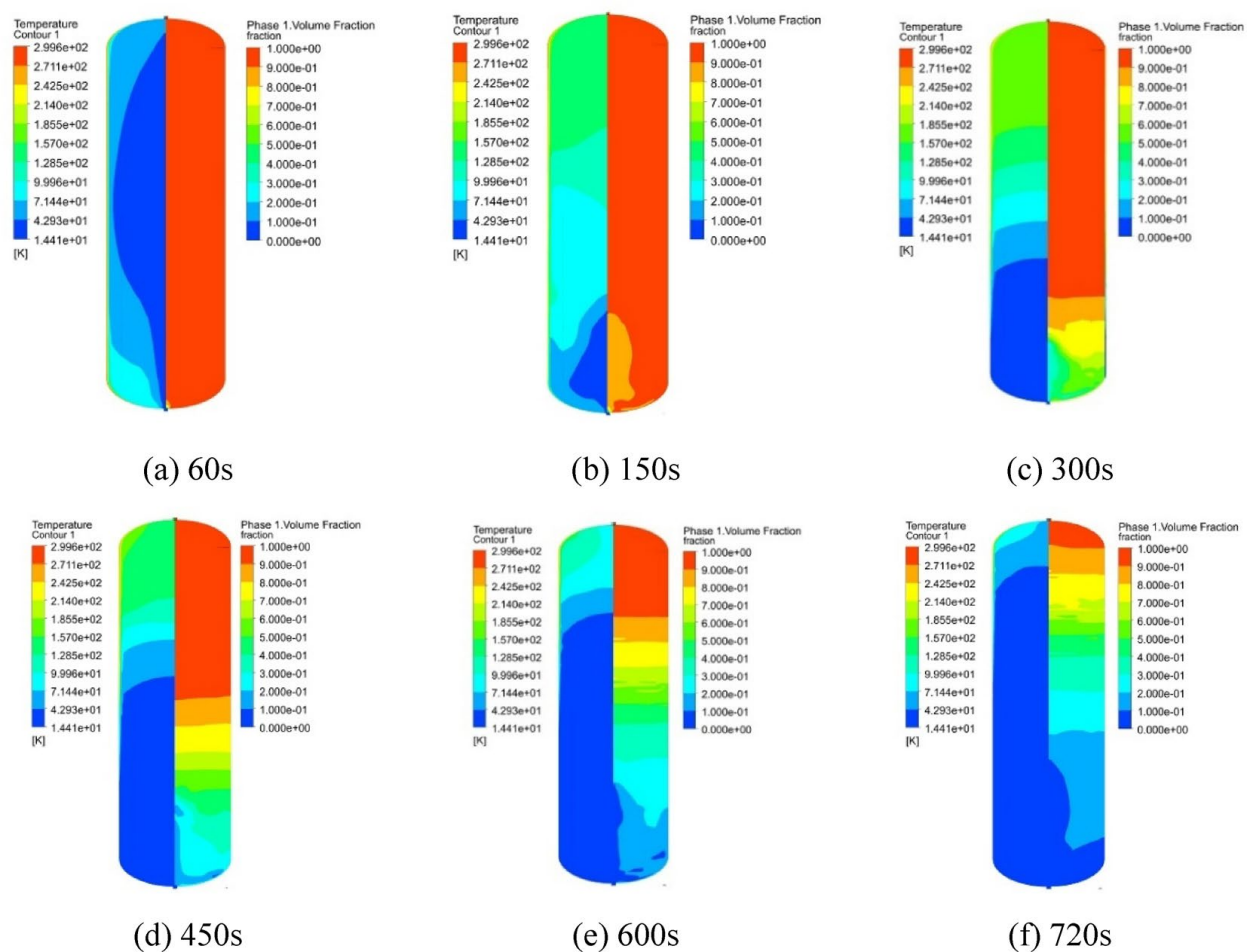
Temperature and two-phase flow field at the pressure-outlet boundary.

The operating conditions are set as follows: a filling flow rate of 8 m³/min, a filling temperature of 14.6 K, a heat transfer coefficient of 20 W/(m·K), and an inlet pressure of 0.1 MPa. The initial environmental temperature is 300 K, and the outlet is configured as a zero-pressure-difference boundary. Figure 4 illustrates the temperature distribution (left half) and gas-liquid phase distribution (right half) on the mid-plane inside the liquid hydrogen tank at different time instants during the filling process. It can be seen from Fig. 2 that the liquid hydrogen volume fraction is 3.5% at t = 60 s, 3.7% at t = 150 s, 13.7% at t = 300 s, 29.1% at t = 450 s, 46.9% at t = 600 s, and 62.4% at t = 720 s. At the initial stage of filling, due to the ambient temperature of 300 K and the absence of a pressure outlet, the liquid hydrogen rapidly vaporizes into hydrogen gas after entering the tank, causing the temperature inside the tank to drop quickly. It can be concluded from the observation of the five monitoring points that the temperature curve shows a trend of first decreasing, then increasing, and then decreasing again. This is because the vaporization of liquid hydrogen slightly reduces the temperature inside the tank in the early filling stage. However, since the ambient temperature is much higher than the liquid hydrogen temperature, the temperature inside the tank returns to close to the initial temperature. With the continuous filling and accumulation of liquid hydrogen, the temperature inside the tank gradually decreases after recovery and approaches the filling temperature. It can be observed from the two-phase flow field diagrams that, owing to the lack of a pressure outlet, the liquid phase does not appear in a stable state at the bottom of the tank until approximately 300 s.

**Fig. 5:**
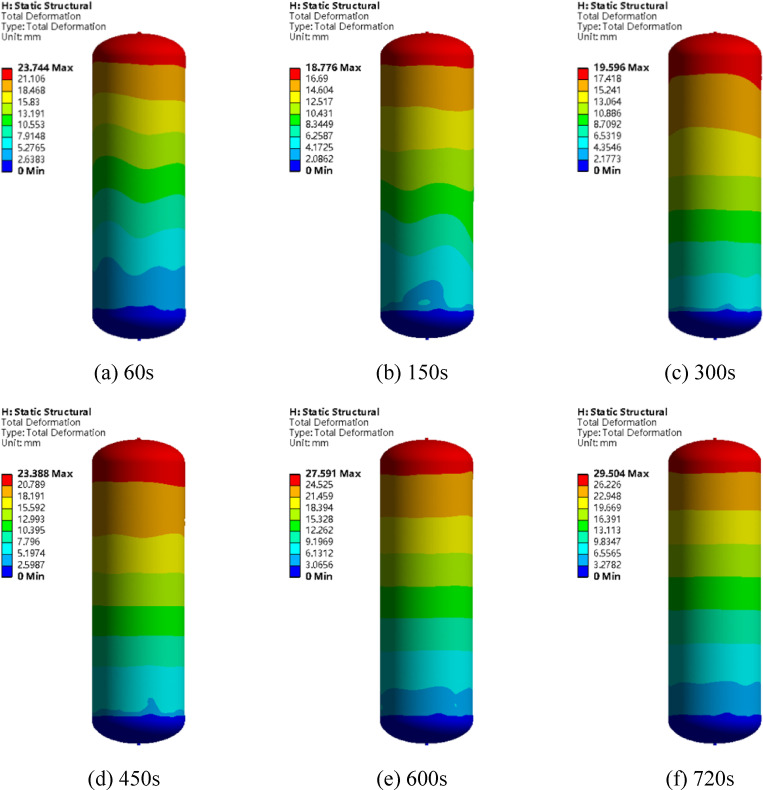
Structural thermal deformation for the zero pressure-difference outlet.

The structural thermal deformation under the condition of no outlet pressure difference is shown in Fig. 5. The deformation is 23.744 mm at t = 60 s, 18.776 mm at t = 150 s, 19.596 mm at t = 300 s, 23.388 mm at t = 450 s, 27.591 mm at t = 600 s, and 29.504 mm at t = 720 s. The deformation gradually increases along the axial direction from bottom to top. Since fixed constraints are applied at the bottom of the tank, the deformation mainly occurs at the upper part of the tank, showing a trend of increasing first, then decreasing, and then increasing again. In the early stage, liquid hydrogen vaporizes into hydrogen gas, leading to a sharp rise in pressure and a rapid drop in temperature inside the tank, which increases the tank deformation. Without a pressure outlet, the liquid phase cannot accumulate rapidly, resulting in a temperature recovery and a decrease in tank deformation during this period. As time increases, the liquid phase in the tank gradually accumulates stably, the tank temperature decreases steadily, and the tank deformation increases gradually.

**Fig. 6:**
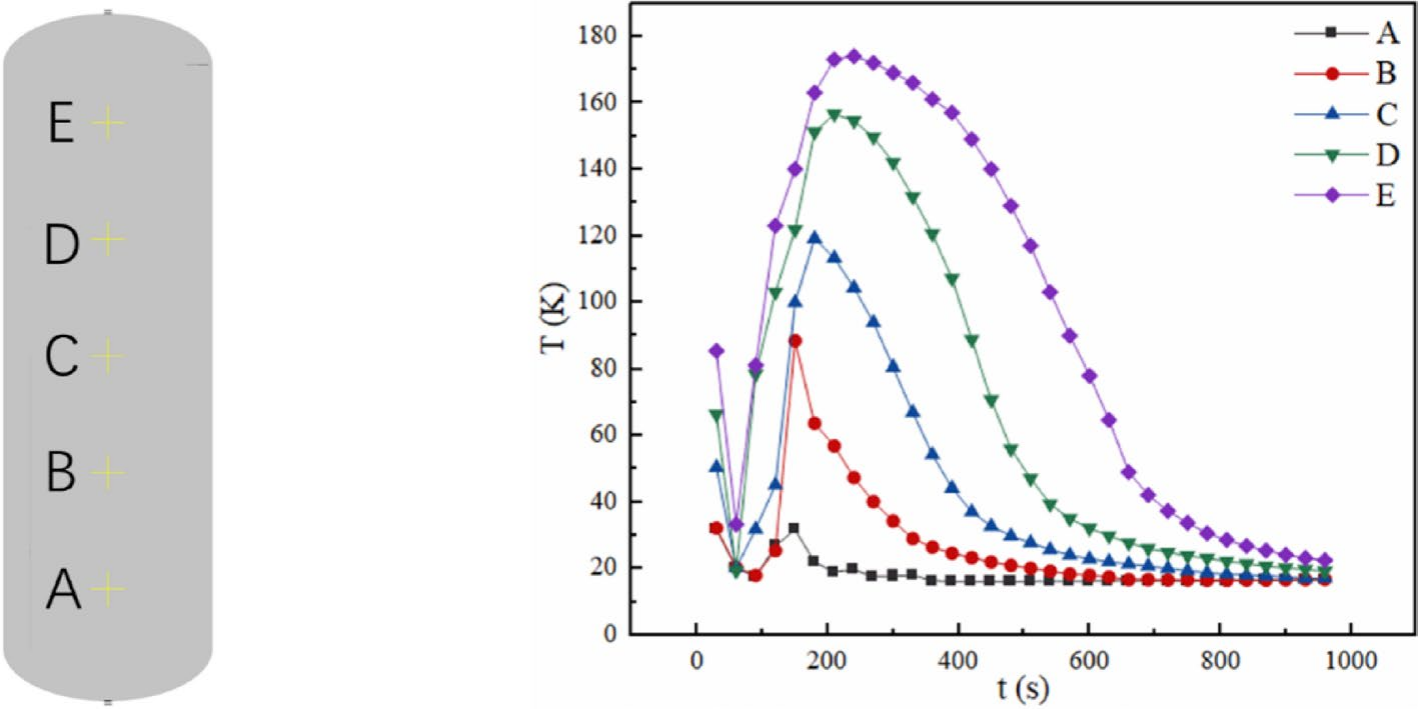
Temperature curve of the reference point on the symmetry axis.

It can be seen from Fig. 6 that the temperature near the bottom of the tank drops first. The temperature decreases briefly from 0 to 100 s, rises between 100 s and 200 s, and then gradually decreases to the filling temperature after 200 s.

**Fig. 7:**
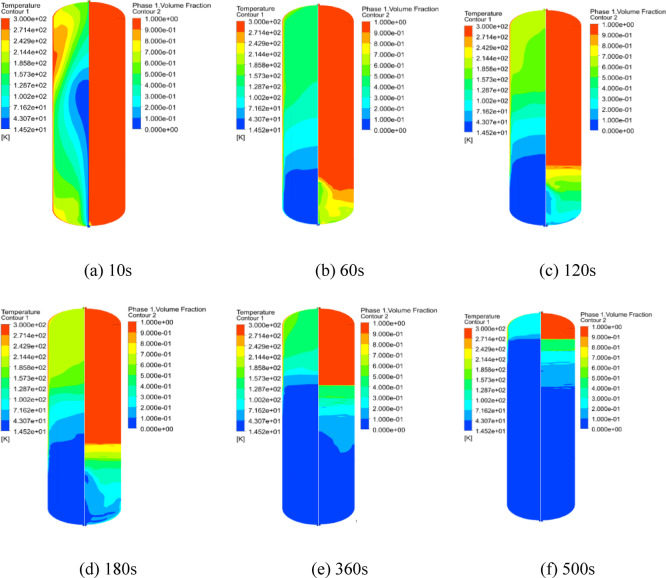
Temperature and two-phase flow field at various instants.

The pressure difference between the inside and outside of the outlet is increased to 0.1 MPa, with all other conditions unchanged. It can be seen from Fig. 5 that, due to the set pressure difference at the outlet, the pressure inside the tank increases as liquid hydrogen vaporizes into hydrogen gas. The liquid hydrogen volume fraction is 3.2% at t = 10 s, 9.3% at t = 60 s, 18.1% at t = 120 s, 27.8% at t = 180 s, 56.3% at t = 360 s, and 78.5% at t = 500 s. The accumulation rate of liquid hydrogen is faster than that under the condition without a pressure outlet (Fig. 7).

**Fig. 8:**
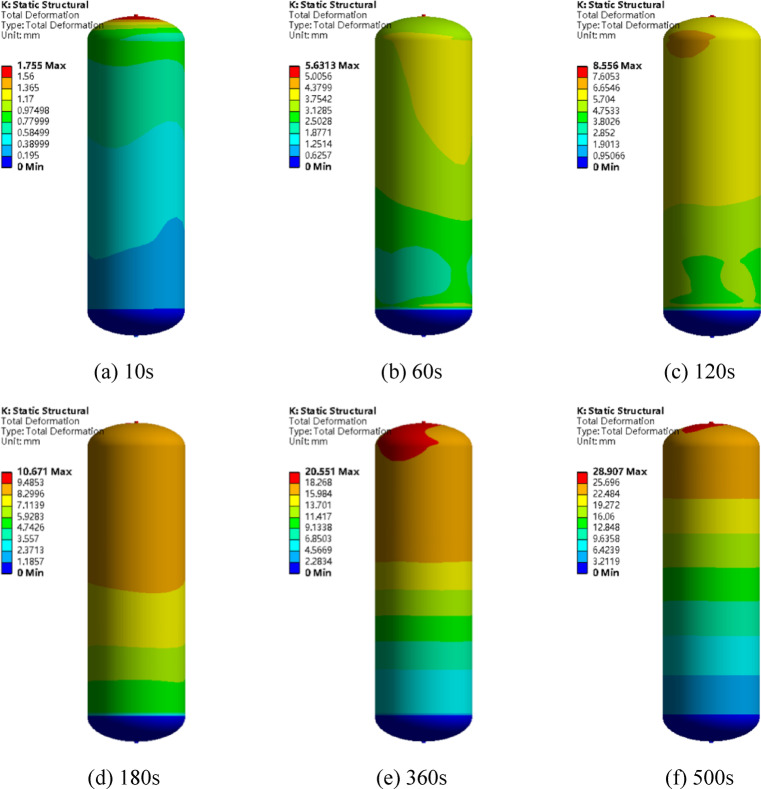
Structural thermal deformation at various instants.

It can be seen from Fig. 8 that due to the pressure difference at the outlet, the overall trend of tank deformation is a gradual increase. The deformation is 1.755 mm at t = 10 s, 5.63 mm at t = 60 s, 8.55 mm at t = 120 s, 10.671 mm at t = 180 s, 20.551 mm at t = 360 s, and 28.907 mm at t = 500 s. At 60 s, the deformation is significantly reduced from 23.744 mm to 5.63 mm compared with the condition without a pressure outlet. The position of maximum deformation is still located at the top of the tank.

**Fig. 9:**
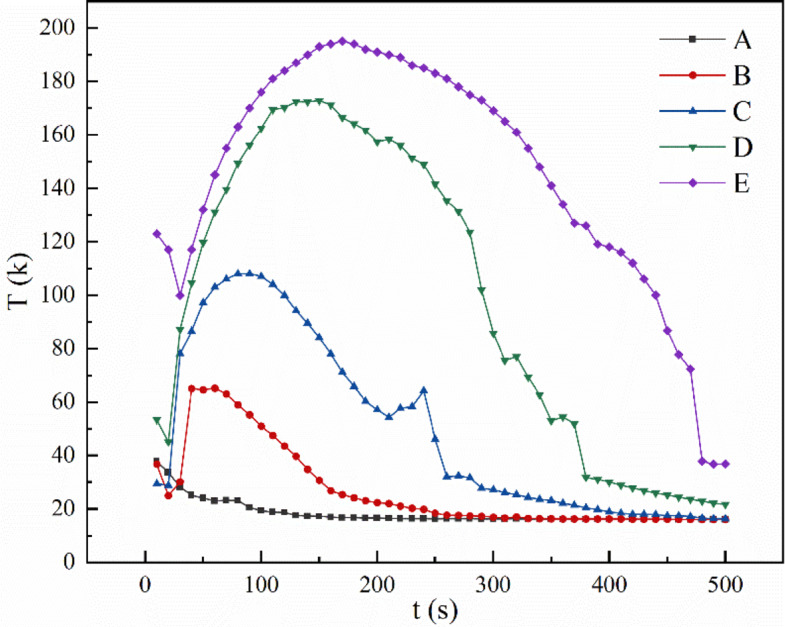
Temperature curve of reference point on symmetry axis.

It can be observed from Fig. 9 that after adding the pressure outlet, the minimum point of each temperature curve is higher than that without a pressure outlet. This is because the pressure outlet increases the pressure inside the tank, resulting in temperature fluctuations, while the overall trend is consistent with that without a pressure outlet.

**Fig. 10:**
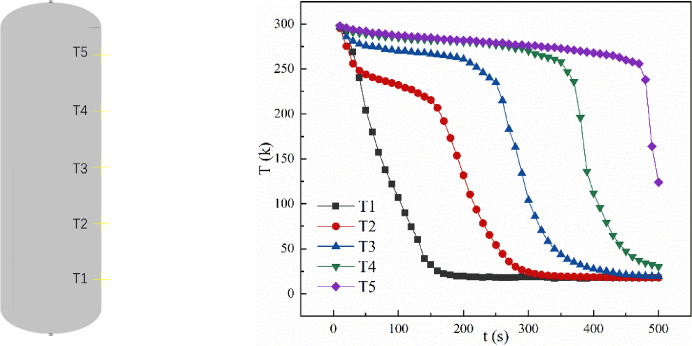
Temperature variation curves of wall reference points.

It can be observed from the wall reference points in Fig. 10 that as the distance from the filling port increases, the temperature trend shows a slow decrease followed by a rapid drop. The temperature variation amplitude of each monitoring point decreases with increasing distance from the filling port. The temperature of T1 drops to the minimum at 150 s, close to the filling temperature. The temperature of T2 decreases rapidly at 150 s and reaches the minimum at 300 s. The temperature of T3 drops sharply at around 250 s and reaches the minimum at 400 s. The maximum cooling rate at T1 is 1.69 K / s. Compared with the experimental results of literature^27^ (1.81 K/s), the cooling rate of this paper is close to the experimental results, which shows the effectiveness of the calculation.

**Fig. 11:**
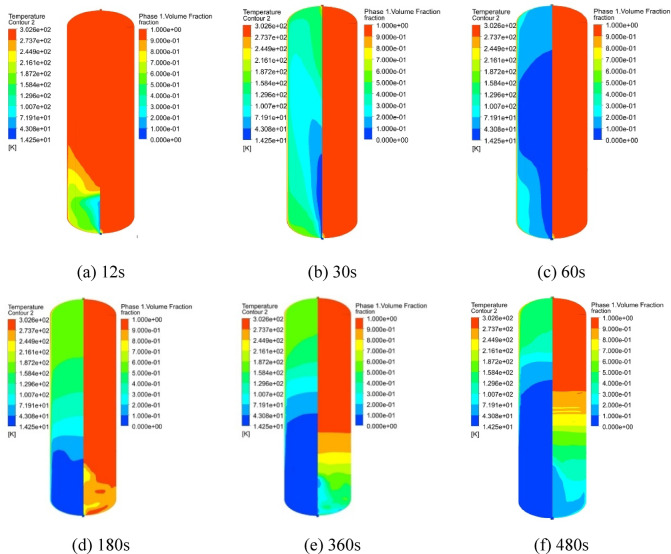
Temperature and two-phase flow field at different instants.

The filling conditions were adjusted to a flow rate of 8 m³/min and an outlet pressure difference of 0.2 MPa to investigate the effect of the outlet pressure difference on the temperature field and structural deformation of the tank. As shown in Fig. 11, the liquid hydrogen volume fraction is 0.6% at t = 12 s, 1.65% at t = 30 s, 3.87% at t = 60 s, 5.52% at t = 180 s, 20.55% at t = 360 s, and 33.13% at t = 480 s. It can be concluded that a larger outlet pressure difference results in a slower accumulation rate of liquid hydrogen.

**Fig. 12:**
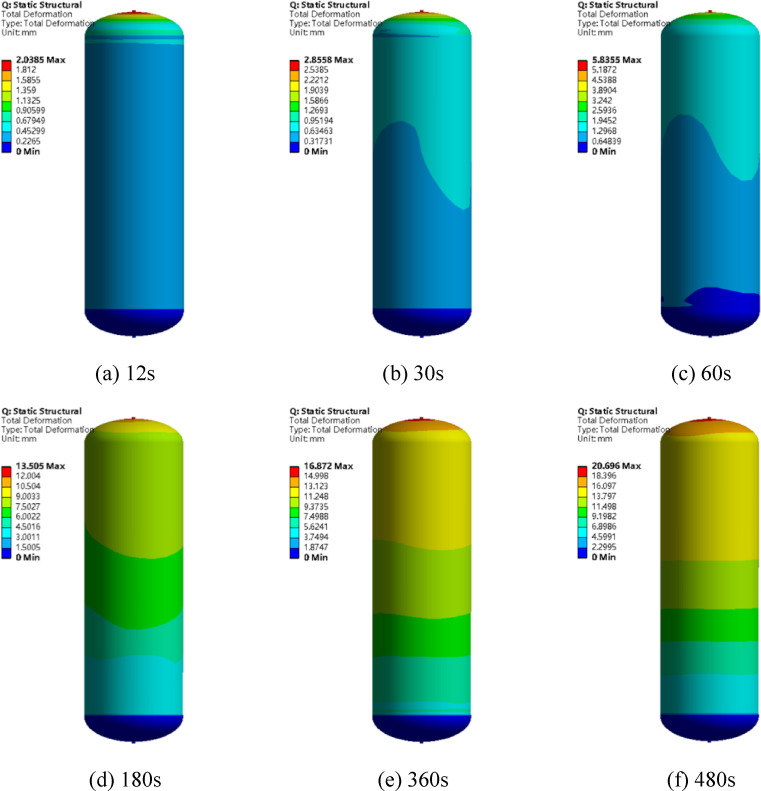
Structural thermal deformation at different instants.

As shown in Fig. 12, the deformation is 2.03 mm at t = 12 s, 2.85 mm at t = 30 s, 5.83 mm at t = 60 s, 13.505 mm at t = 180 s, 16.872 mm at t = 360 s, and 20.696 mm at t = 480 s. Compared with the deformation under 0.1 MPa, the two exhibit basically the same deformation trend, both increasing gradually, with the maximum deformation occurring at the top. Before 360 s, the deformation is smaller under a lower outlet pressure, while after 360 s, the deformation under a higher outlet pressure exceeds that under a lower outlet pressure.

**Fig. 13:**
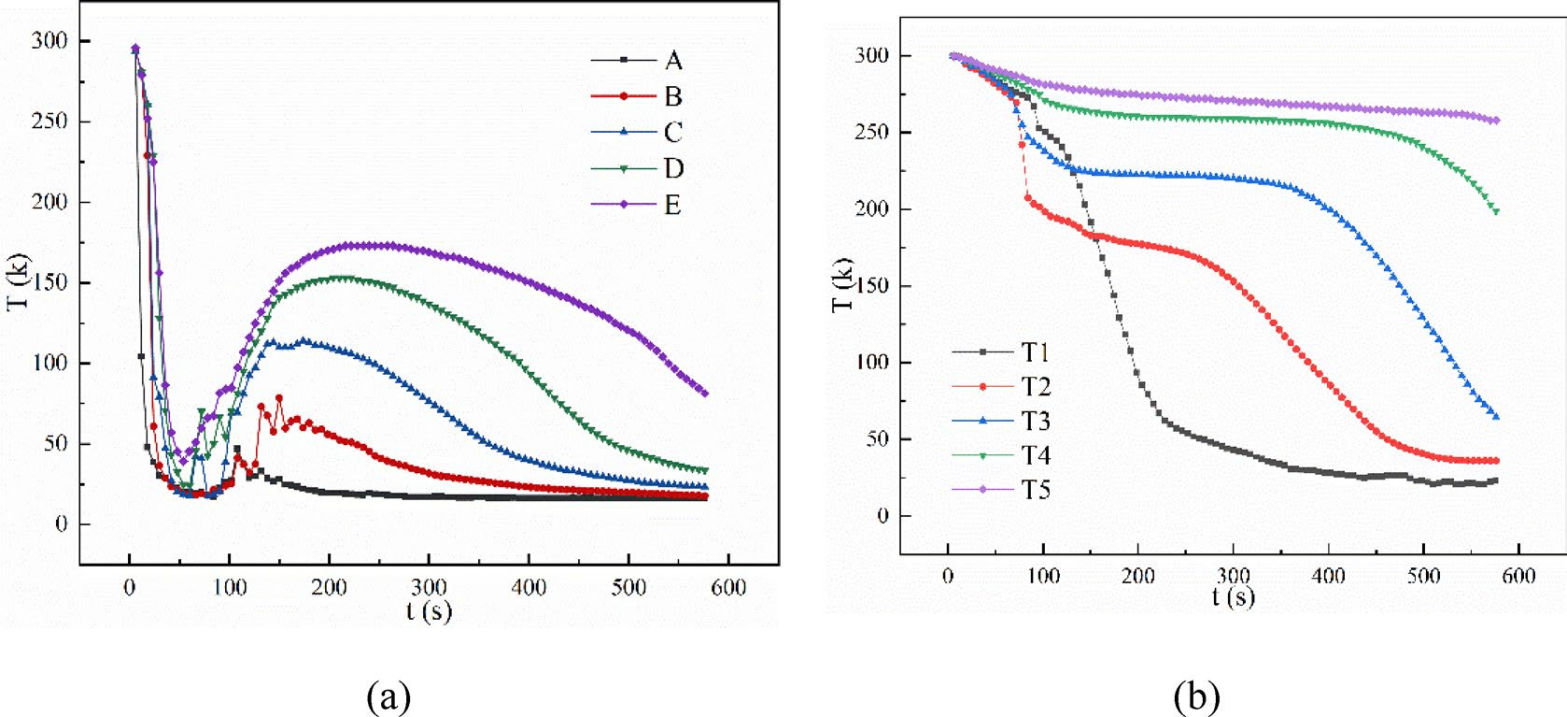
Reference point temperature curves.

As shown in Fig. 13, when the outlet pressure difference is increased to 0.2 MPa, the peak rebound temperature under the same conditions is larger than that at 0.1 MPa, and the liquid phase accumulation occurs earlier than that at 0.1 MPa. After temperature rebound, the temperature decreases more slowly than that at 0.1 MPa. When the outlet pressure difference increases to 0.2 MPa, the wall temperature decreases slowly within 0–100 s. After 100 s, T1, T2, and T3 show a sharp decreasing trend, while T3, T4, and T5 change gently, and then decrease steadily to the filling temperature. Compared with the inflection point under the condition of 8 m³/min and 0.1 MPa, the inflection point in the final stage appears later. When the outlet pressure difference is increased to 0.2 MPa, the peak rebound temperature under the same conditions is larger than that at 0.1 MPa, and the liquid phase accumulation occurs earlier than that at 0.1 MPa. After temperature rebound, the temperature decreases more slowly than that at 0.1 MPa.

**Fig. 14:**
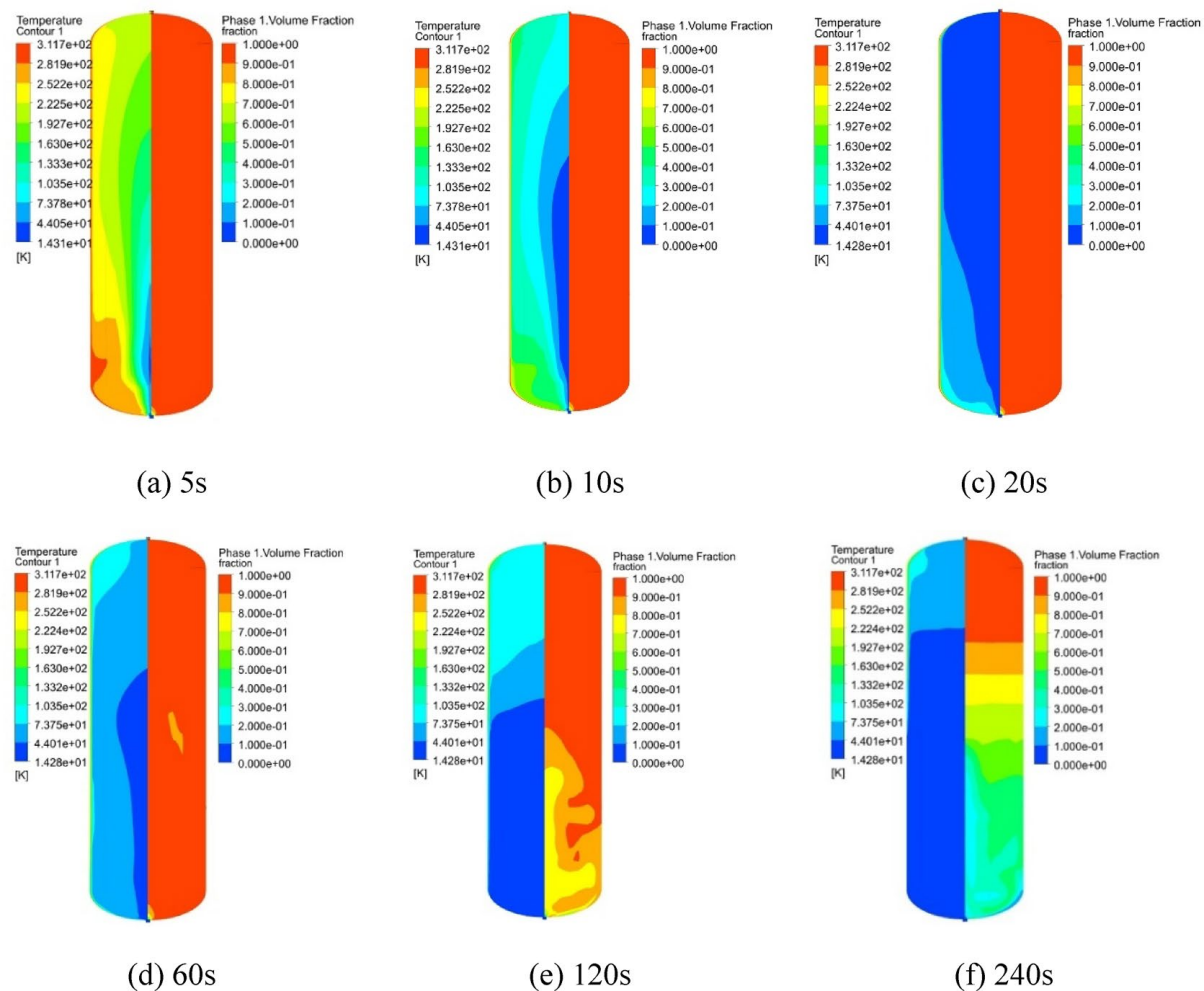
Flow field temperature and two-phase distribution at different instants.

The flow rate was increased to 15 m³/min with the outlet pressure maintained at 0.1 MPa, and all other boundary conditions remained consistent with the initial settings to investigate the relationship between flow rate and the flow field and stability inside the thin-walled tank. As shown in Fig. 12, the temperature near the filling port rapidly drops to the inlet temperature of 14 K. Over time, the low-temperature region expands upward and laterally. Although the top of the tank can reach the low temperature of 14 K at around 20 s, the temperature at the top rises again due to flow oscillation and then stabilizes in the subsequent filling stage. At 240 s, the gas-liquid interface becomes stable, and the temperature distribution also stabilizes accordingly. It can be observed from Fig. 12 that with the flow rate increased to 15 m³/min, the outlet pressure maintained at 0.1 MPa, and all other boundary conditions unchanged, the increased flow rate significantly shortens the overall filling time. The central region of the tank cools down first, and the temperature distributes along the filling direction and radial direction (Fig. 14). The liquid hydrogen volume fraction is 1.27% at t = 5 s, 2.55% at t = 10 s, 5.23% at t = 20 s, 4.275% at t = 60 s, 12.3% at t = 120 s, and 40.04% at t = 240 s.

**Fig. 15:**
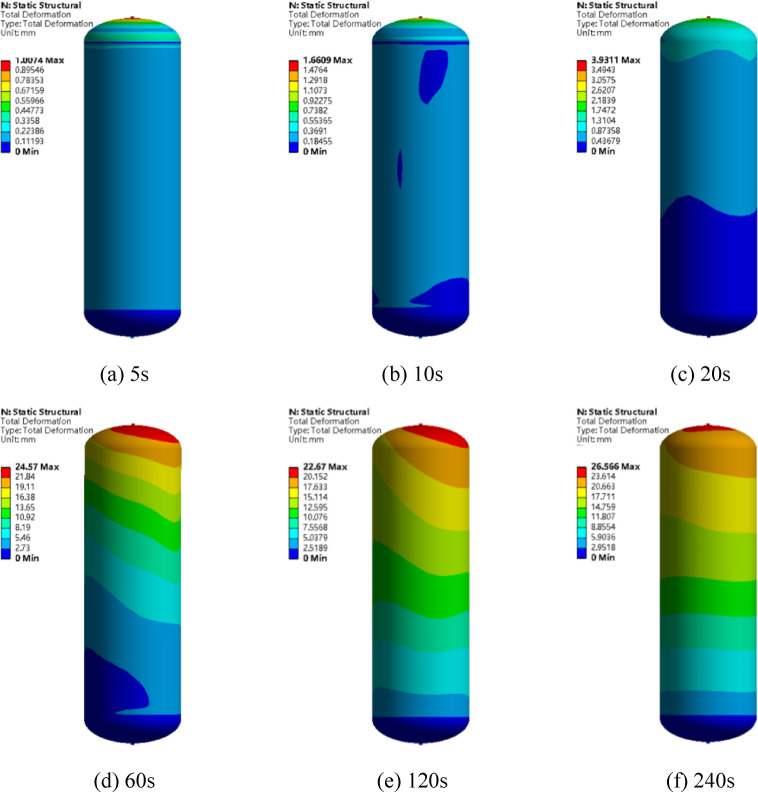
Structural thermal deformation at different instants.

**Fig. 16:**
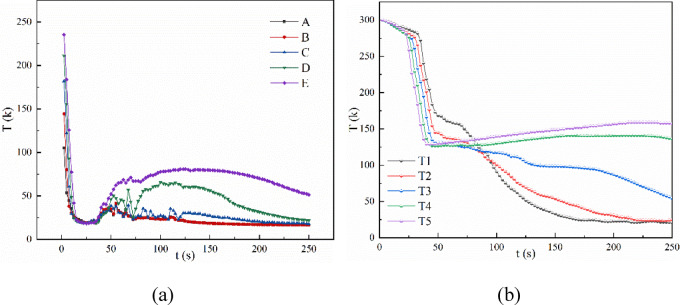
Temperature variation curves of reference points (15m^3^/min).

As shown in Fig. 15, at the initial stage, the temperature is relatively high due to the just-injected low-temperature fuel, resulting in relatively small tank deformation. The deformation is 1 mm at t = 5 s, 1.66 mm at t = 10 s, 3.93 mm at t = 20 s, 24.57 mm at t = 60 s, 22.67 mm at t = 120 s, and 26.57 mm at t = 240 s. Compared with the condition of 8 m³/min and 0.1 MPa outlet pressure, the deformation is 1.755 mm at t = 10 s, 5.63 mm at t = 60 s, and 8.55 mm at t = 120 s. With the decrease of tank temperature, the deformation increases rapidly, accompanied by periodic oscillations, and finally stabilizes. As the flow rate increases, the filling time of the same tank is shortened accordingly. The temperature inside the tank drops rapidly within 10 s, and no liquid hydrogen accumulates in the first minute of filling. The temperatures at the five monitoring points inside the tank are shown in Fig. 16. It can be seen that the overall trend of the five points is first decreasing, then slightly increasing, and finally approaching the filling temperature. Compared with the low flow rate (8 m³/min), the peak of temperature rebound is much lower than those under no pressure outlet and low flow rate (8 m³/min) conditions. This is because the increased flow rate raises the volume fraction of evaporated liquid hydrogen, which absorbs more heat inside the tank under pressurized conditions. Meanwhile, the pressure outlet allows the evaporated liquid hydrogen to fully absorb heat inside the tank, resulting in a lower rebound temperature than that without a pressure outlet.

It can be seen from Fig. 16 that the wall temperature decreases slowly within 0–25 s, and all five points reach about 275 K at 25 s, after which the temperature drops rapidly. When the filling time reaches 50 s, the variation of each point slows down. Since T4 and T5 are far from the filling port, their temperatures rise slightly, with the minimum temperature of these two points being about 125 K. The other three points show an increasing cooling rate after slow cooling, and finally approach the filling temperature. The core energy exchange in the process of liquid hydrogen filling occurs between the high temperature wall and the low temperature liquid hydrogen, which mainly involves two forms of energy transfer : convective heat transfer and latent heat of vaporization. In the initial stage of filling, the temperature difference between the wall and the liquid hydrogen is very large, and the convective heat transfer is very strong, resulting in a rapid decrease in the wall temperature. Heat is transferred from the wall with higher temperature to the fluid with lower temperature by convection. As the wall temperature decreases, the temperature difference decreases, and the convective heat transfer rate also decreases. After absorbing heat, liquid hydrogen undergoes a phase change from liquid to gas. This process requires absorbing a large amount of heat, namely latent heat of vaporization, which is the main energy absorption mechanism of the cooling wall. In the early stage of filling, most of the heat transferred from the wall is used to vaporize the liquid hydrogen, rather than directly increasing the fluid temperature, so the wall temperature can decrease rapidly. At this time, due to the continuous injection of liquid hydrogen, further heat transfer is hindered. When the input heat is greater than the heat absorbed by liquid hydrogen vaporization, the wall temperature begins to rise. As a result, the back temperature of 15m^3^/min in the large flow filling condition is lower than that of 8m^3^/min in the filling condition.

**Fig. 17:**
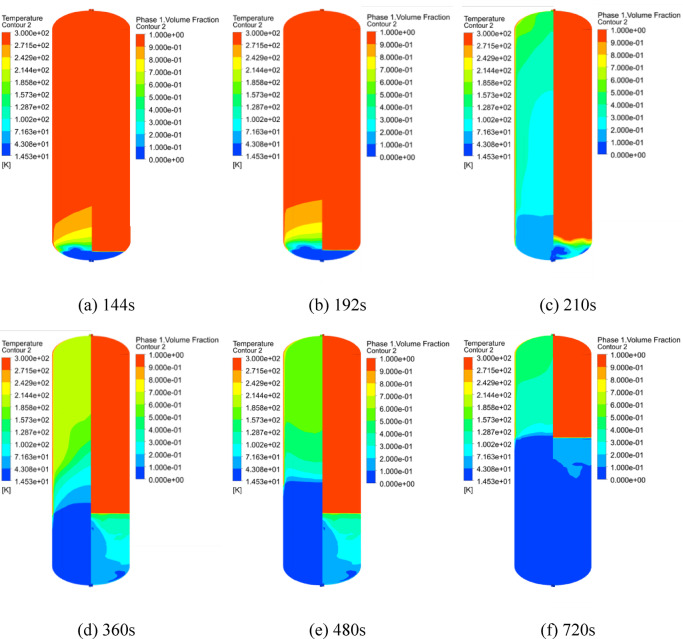
Flow field temperature and two-phase schematic diagram.

Precooling is divided into two stages. The first stage is low-flow-rate filling with a velocity of 2 m/s, filling 5% of the total volume. In the second stage, the flow rate is increased to 8 m³/min to investigate the flow field temperature and deformation. It can be observed from Fig. 17 that, due to the filling velocity of 2 m/s during the precooling stage, the overall temperature changes little after precooling, with only a small amount of liquid hydrogen accumulating at the bottom of the tank. After switching to high-flow-rate filling, the tank temperature changes significantly and the liquid hydrogen volume fraction increases greatly. The liquid hydrogen volume fraction is 4.99% at t = 144 s, 4.99% at t = 192 s, 9.29% at t = 210 s, 24.975% at t = 360 s, 35.42% at t = 480 s, and 55.05% at t = 720 s. At 210 s, the maximum temperature in the flow field is about 214 K and the minimum temperature is about 43 K. At 360 s, the maximum temperature remains about 214 K, while the region near the filling temperature expands.

**Fig. 18:**
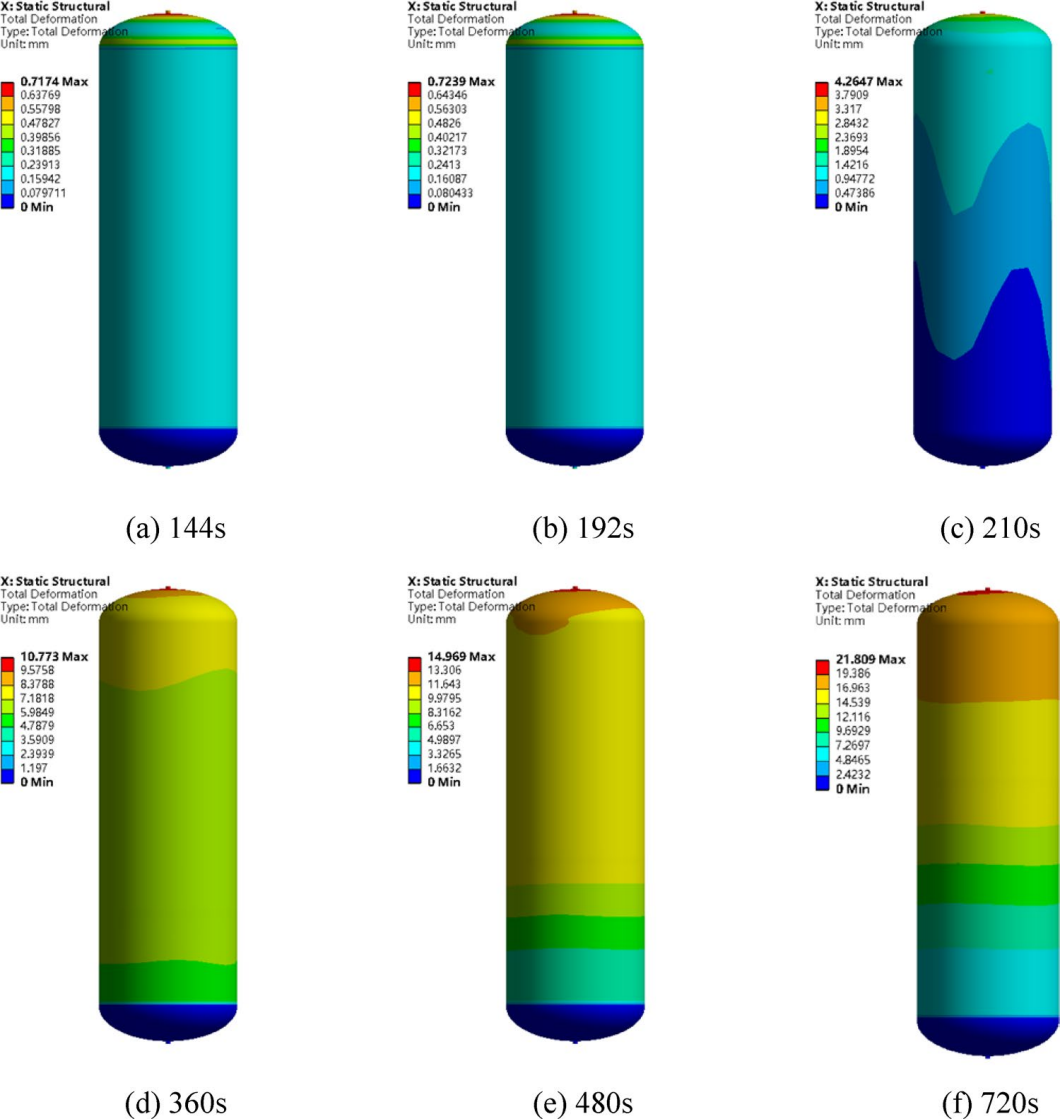
Structural thermal deformation diagram.

As shown in Fig. 18, the deformation is 0.717 mm at t = 144 s, 0.72 mm at t = 192 s, 4.26 mm at t = 210 s, 10.77 mm at t = 360 s, 14.96 mm at t = 480 s, and 21.8 mm at t = 720 s. The precooling stage lasts 192 s, during which the tank deformation is very small because the filling velocity is only 2 m/s. The deformation generated in this process is thermal strain caused by liquid hydrogen absorbing heat. After 192 s, as the filling rate increases, the tank deformation gradually increases. However, due to precooling, the tank deformation is much smaller than that without precooling under the same working conditions.

**Fig. 19:**
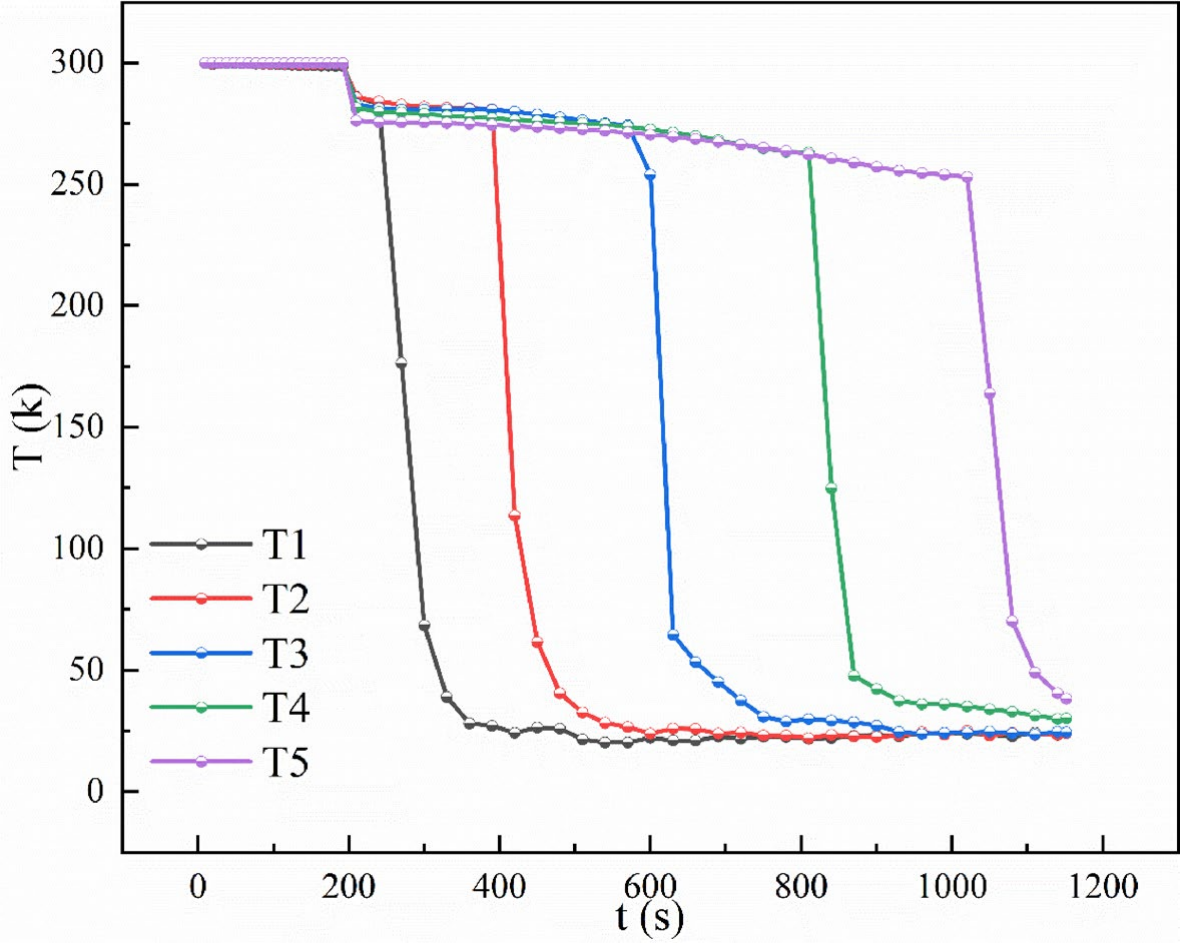
Temperature curves of wall reference points under precooling conditions.

It can be seen from Fig. 19 that during the initial precooling stage, the flow rate is very low, resulting in little change in the flow field temperature. After switching to a high flow rate, the temperatures at the five wall points decrease successively and approach the filling temperature.

## Conclusions

1. When the flow rate is 8 m³/min and the pressure difference is 0, it takes 960 s for the tank to be filled with 84.1% fuel, and the deformation is 30.418 mm. When the outlet pressure difference increases from 0 to 0.1 MPa, it takes 500 s to fill 78.5% fuel, with a deformation of 28.907 mm. When the outlet pressure difference increases from 0.1 MPa to 0.2 MPa, it takes 576 s to fill 45% fuel, with a deformation of 24.527 mm. The increase in outlet pressure difference leads to longer fuel filling time and lower deformation.
2. When the flow rate is increased to 15 m³/min, it takes 250 s to fill 44% fuel, and the deformation is 27.043 mm. The efficiency of liquid hydrogen filling at high flow rate is significantly higher than that at low flow rate, and the deformation is larger than that under low flow rate.
3. When precooling is adopted, the tank deformation after switching to high-flow-rate filling is smaller than that without precooling. Precooling can greatly improve the efficiency of liquid hydrogen filling and significantly reduce the thermal deformation of the structure.

## Data Availability

The datasets generated and/or analysed during the current study are not publicly available due to time limitations but are available from the corresponding author on reasonable request.
